# Circadian Models of Serum Potassium, Sodium, and Calcium Concentrations in Healthy Individuals and Their Application to Cardiac Electrophysiology Simulations at Individual Level

**DOI:** 10.1155/2013/429037

**Published:** 2013-09-03

**Authors:** Kamil Fijorek, Miroslawa Puskulluoglu, Sebastian Polak

**Affiliations:** ^1^Department of Statistics, Cracow University of Economics, 27 Rakowicka Street, 31-510 Krakow, Poland; ^2^Department of Oncology, Jagiellonian University Medical College, 20 Grzegorzecka Street, 31-531 Krakow, Poland; ^3^Unit of Pharmacoepidemiology and Pharmacoeconomics, Faculty of Pharmacy, Jagiellonian University Medical College, Medyczna 9 Street, 30-688 Krakow, Poland

## Abstract

In the article a brief description of the biological basis of the regulation of human biological clocks was presented in order to introduce the role of circadian rhythms in physiology and specifically in the pharmacological translational tools based on the computational physiology models to motivate the need to provide models of circadian fluctuation in plasma cations. The main aim of the study was to develop statistical models of the circadian rhythm of potassium, sodium, and calcium concentrations in plasma. The developed ion models were further tested by assessing their influence on QT duration (cardiac endpoint) as simulated by the biophysically detailed models of human left ventricular cardiomyocyte. The main results are model equations along with an electronic supplement to the article that contains a fully functional implementation of all models.

## 1. Introduction

The crucial role of homeostasis maintenance in all living creatures is not in contradiction with the observation that various biological parameters are not static. The rhythmical changes observed in humans that occur periodically play an important role in the adaptation to the dynamic environment. Chronobiology influences the activity and functions of organs and tissues and is also a driver of anatomical, physiological, and molecular changes. Classification of biological rhythms depends on interval duration, starting with the very short periods expressed in seconds (e.g., electrocardiographic changes), through ultradian periods described in minutes/hours (e.g., sleep), and circadian periods close to 24 hours, up to longer periods, including monthly (circatrigintan, i.e., menstrual) and yearly (circannual) rhythms [1]. It has been suggested that in humans, the physiological rhythmicity and its behavioral reflection define chronotype (e.g., morningness versus eveningness tendencies).

In the following sections, a brief description of the biological basis of the regulation of human biological clocks is presented in order to introduce the role of circadian rhythms in physiology and specifically in the pharmacological translational tools based on the computational physiology models to motivate the need to provide models of circadian fluctuation in the main plasma cations.

## 2. Human Biological Clocks

The center of the circadian clock is localized in the bilateral suprachiasmatic nuclei (SCN) in the hypothalamus [1–4]. Also, organs known as peripheral circadian oscillators, for example, the heart, liver, kidneys, are thought to be responsible for circadian rhythmicity of human physiology, behavior, or biochemistry. Information exchange between clocks involves humoral and nervous systems and includes feedback loops. Peripheral oscillators may also show their autonomous nature [2–6]. There are many genes that are expressed in a clock-dependent manner in both central or peripheral clock tissues—for details see numerous reviews [1–3]. As for the cardiovascular system, circadian variation may be seen in blood pressure, heart rate, coronary blood flow, hormonal secretion, or electrical activity [2, 7–10]. The clock localized in the heart influences the cells' response to numerous physiological or pathological conditions, for example, ischemia [11, 12]. Examples of external and internal factors influencing heart functioning are physical activity and autonomic nervous/humoral system (e.g., glucocorticoids, renin-angiotensin-aldosterone activity) modulating blood pressure, for example, causing its increase in the morning [2, 3]. However, most studies on peripheral clock localized in cardiomyocytes and factors influencing it were performed on animals. Although comprehensive data on humans are still lacking, it was shown that almost 10% of genes in heart muscle tissue are expressed in a clock-dependent manner [2, 9]. Thus, the control and the loops between the clocks take place on different levels: from organs and systems through biochemical, cellular to molecular levels. 

It is not only the physiological functioning of the heart, but also many diseases such as atrial fibrillations and other arrhythmias (also those triggered by drugs), myocardial infarction, or sudden cardiac death that show circadian variation [5, 7, 13]. The incidence of most adverse cardiological events is related to the time of the day [1, 5]. Moreover, the studies suggest that circadian oscillation (e.g., RR or QT interval, R and T wave voltage) may play a predicting role in cardiac death. It was shown that prolongation of the QTc interval plays a prognostic role and also reduced circadian fluctuations are connected with poorer patients' outcome [2, 13].

The biological rhythmicity can be influenced by genetic factors and is frequently related to the risk of developing a number of diseases and their further course [3, 14–16]. Therefore, chronotype modification has been used as a therapeutic strategy [17] for various diseases including cardiovascular disturbances [2].

## 3. Chronopharmacology

Circadian rhythms have been actively investigated and applied in clinical pharmacology. The accumulation of knowledge in this area led to the formation of a new field called chronopharmaceutics, which aims at delivering drugs in a controlled manner at the most appropriate time of the day [18], which in turn leads to the optimization of drugs dosing and their clinical effect. Various drugs and populations were investigated in chronopharmacology research; for example, see Block et al. [19]. Numerous studies also confirmed that circadian rhythms influence both the pharmacokinetics and pharmacodynamics of drugs. Time of drug application modifies drug pharmacokinetics by changing all the elements of the ADME pathway—absorption, distribution, metabolism, and excretion [20]. As a result, many drug pharmacokinetic parameters are influenced to some degree, which in turn impacts drug efficacy and safety, that is, the pharmacodynamic components of chronopharmacology [21–23].

## 4. Cardiac Electrophysiology Simulations

Chronopharmaceutics, however, is not the only area of pharmaceutical sciences that can benefit from research into the human rhythms. Translational tools based on the biophysically detailed mathematical models allowing for *in vitro-in vivo* extrapolation are gaining increasing interest and are more and more commonly used in systems pharmacology [24]. In cardiology, simulations based on the models of cardiac electrophysiology are utilized for the assessment of drugs' cardiac safety [25, 26]. Approaches taken by different research groups vary significantly with respect to the modelling techniques, level of models complexity, heart representation (ranging from single cell up to the two- and whole heart three-dimensional simulations), and the simulated endpoints [27–29]. Regardless of the applied methodology, most of the published manuscripts report results nonspecific to any individual patient by only using simulations constant (usually average) values of parameters describing human biology [30]. Based on the publicly available data sources, empirical models describing the distribution of each relevant physiological parameter in populations of human individuals can be developed and introduced into cardiac electrophysiology simulations. For example, in Polak et al. [31] and Polak and Fijorek [32], regression models relating age to the volume of cardiomyocyte and cell electric capacitance were developed and later used to introduce interindividual variability into the cardiac electrophysiology simulations. In Fijorek et al. [33] inter- and intraindividual variability was introduced into the cardiac electrophysiology simulations by accounting for circadian rhythmicity of the heart rate. However, the circadian rhythmicity of many other physiological parameters can be similarly modeled and introduced into the simulations.

## 5. Study Aims

The main aim of the study was to develop statistical models of the circadian rhythm of three plasma ions, that is, potassium, sodium, and calcium. The developed ion models were further tested by assessing their influence on QT duration (cardiac endpoint) as simulated by the biophysically detailed models of human left ventricular cardiomyocyte. The relevance of the ions to cardiac electrophysiology was described previously [34].

## 6. Materials, Methods, and Results

The first stage included estimation of mean and standard deviation of concentration of potassium, sodium, and calcium. Suitable data was obtained from Polak et al. [34], which is an archive of data on plasma concentration of potassium, sodium, and calcium extracted from scientific articles via extensive literature search. The data were available for a large number of healthy subjects of both sexes (496, 553, and 475 males; 328, 322, and 1783 females; potassium, sodium, and calcium, resp.) and a wide range of ages (4–69, 4–103, and 2–75).

Estimation of mean concentration and standard deviation of concentration (Table 1) was performed separately for both sexes since statistically significant differences between them were found. Different normal ranges of metabolic panel components for men and women are already used in clinical practice for parameters such as: liver function tests, uric acid, creatinine, creatine kinase, and hormone levels [35, 36]. However, for potassium, sodium, and calcium, separate normal ranges for men and women have not been used so far in daily clinical practice.

Markowitz et al. [37] suggested the existence of a relationship between age and ions concentration. However, the results of meta-analysis presented in Polak et al. [34] show no significant age dependence for potassium and sodium concentration. In the case of calcium, there might be a weak positive relationship with age; however, the age impact seems to be too small to be of clinical relevance in the context assumed in this paper, but further research in this area is needed. As a result, none of the developed models include age as an explanatory variable.

There is a noticeable evidence of the existence of a circadian rhythm in potassium and sodium ion concentrations [38–40]. Kanabrocki et al. [41] additionally studied a circadian rhythm in calcium ion concentration; however, the results were not statistically significant, probably due to the rather small number of subjects included in the study. Given insufficient support for calcium rhythmicity, the model developed for that ion does not contain a circadian component.

For potassium and sodium, it was decided to base the new models on the models proposed by Sennels et al. [38] after they had been extensively modified. The main argument in favor of the Sennels' model is that among the existing models, this is the one with the largest number of subjects used for its development (23 individuals, all men). Additionally, it is the most recently published model, so its participants are believed to be the most similar to the presently living population of healthy people. Also, the samples were taken 9 times for every individual (at 9:00, 12:00, 15:00, 18:00, 21:00, 0:00, 3:00, 6:00, and 9:00), which surpasses any other reviewed report, giving Sennels' study more power to detect a circadian rhythm. The standard deviation of residuals (the potassium and sodium model) was estimated from the data extracted from the box plots presented in Sennels' paper using the method described by Hozo et al. [42]. The male and female mean concentrations were taken from Table 1 and incorporated into the developed models. Due to the lack of suitable data, it was assumed that the circadian rhythm is the same for men and women.

In the case of calcium, it was assumed that the concentration values between 2.0–2.8 mM are physiologically valid [34]. Consequently, the assumption of normality of calcium concentration was dismissed, and logit-normality was assumed; that is, concentration is normal after a scaled logit transformation. After transformation, mean calcium concentration for males was 0.1 (SD = 1.3) and for females −0.5 (SD = 0.8). In the case of the potassium and sodium model, such transformation was not needed since both models conformed with the physiological concentration ranges satisfactorily (residuals in both models were assumed to follow the normal distribution). Having completed the above-mentioned stages, the ionic means are calculated by the following formulas:

(1) mean  potassium  =(M/F  mean  concentration)   +0.18∗COS(2∗PI24∗(Time  —10:07)),mean  sodium  =(M/F  mean  concentration)   +1.1∗COS(2∗PI24∗(Time  —13:08)),mean  logit-transformed  calcium  =(M/F  mean  logit-transformed  concentration),

where M/F—male or female; time—value from 0 to 24 range.

In the next phase, the stochastic part of the models was extended by incorporating a physiology-based assumption that for a given individual, the closer in time the concentrations are measured, the more similar are the deviations from the mean concentration. The most common way to induce a correlation between residuals is to use an autoregressive process. In order to estimate the parameters of this process, concentration trajectories from individual subjects are needed. These were extracted for potassium from the Kanabrocki et al. [43] and Williams et al. [44] papers, for sodium from the Kanabrocki et al. [43] paper, and for calcium from the Kanabrocki et al. [43], Jubiz et al. [45], and Wills [46] papers. The data were used to estimate the autocorrelation coefficient of autoregressive process of order one—AR(1). In all of the enumerated studies, the number of subjects was very small, and ionic concentrations were evaluated very sparsely. Consequently, it was found that the data was not able to support a more sophisticated autoregressive model. Results for the AR(1) process are presented assuming a 15-minute sampling interval for ionic concentrations (Table 2).

**Figure 1:**
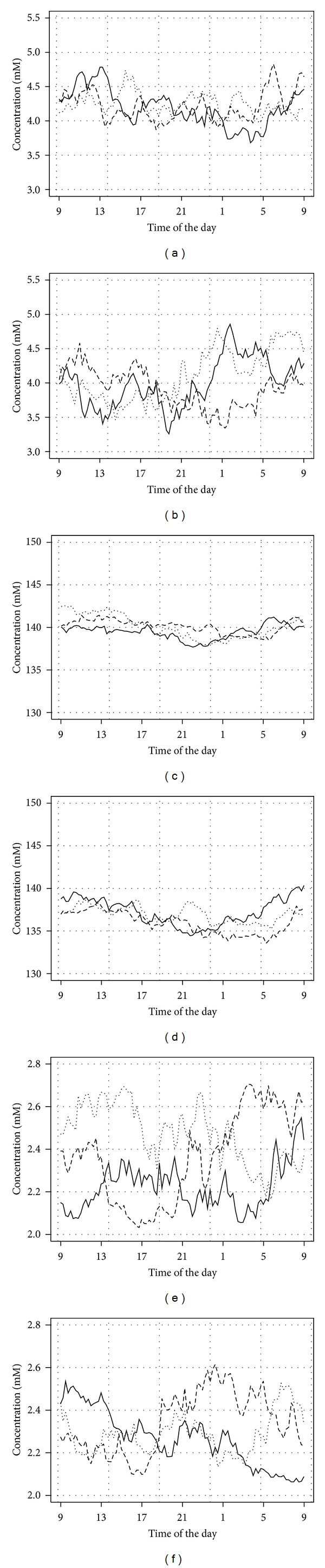
Sample random paths of ionic concentrations: (a) potassium male, (b) potassium female, (c) sodium male, (d) sodium female, (e) calcium male, and (f) calcium female.

In the next stage, the validity of the developed models was verified. A set of a 1000 random model paths of ionic concentrations for males and females for each ion was generated.  Figure 1 shows 3 random paths of each kind. The paths successfully reproduced the theoretical properties of the models as given by the models equations and estimated coefficients; that is, generated data showed circadian rhythmicity (excluding the calcium model) and the sex-dependent mean level and variability, also the generated concentrations were within physiological ranges. Consequently, models were deemed internally valid. An electronic supplement to the paper contains a fully functional Excel implementation of all the described models.

As computer models of heart physiology become increasingly popular as translational tools utilized, for example, for the drugs' proarrhythmic potency assessment, proper and detailed account of the physiological parameters' inter- and intraindividual variability becomes an element of the greatest importance. It should therefore be suggested that circadian rhythms are an important element of human biological variability. Consequently, in the last stage, the developed models were built in to the ToxComp platform simulating the cardiac electrophysiology at the cellular (single cardiomyocyte) and heart wall (1D strand) level. The ToxComp platform combines a physiologically based electrophysiological model of human left ventricular cardiomyocytes and a database of human physiological, genotypic, and demographic data enabling the prediction of the QT prolongation in humans based on the *in vitro* data [25, 31]. To account for the heterogeneities in ionic currents between endocardial, midmyocardial, and epicardial cells, 1D strand paced at the epicardial side was constructed. The 50 : 30 : 20 distribution of the endo-, mid-, and epicardial cells was used together with a diffusion coefficient equal to 0.0016 cm^2^/ms. The Forward Euler method was used to integrate model equations. Integration results were used to calculate a pseudo-ECG. First and last beats were excluded from the simulated pseudo-ECG traces. A space step and a time step were set to Δ*x* = 0.01 mm and Δ*t* = 0.01 ms. 100 virtual individuals were simulated (50% of females) in the age range of 18–55 (mean = 32.7, SD = 9.0). The values of the nonionic physiological parameters were randomly assigned to each virtual individual to mimic population consisting of healthy volunteers as previously described [31]. For every virtual individual, a 10 000 milliseconds long pseudo-ECG traces were simulated at the following 12 time points during the day: 6:00, 8:00, 10:00, 12:00, 14:00, 16:00, 18:00, 20:00, 22:00, 0:00, 2:00, and 4:00. The simulations were performed under the assumption of five different scenarios. In the scenario “A,” constants K^+^, Ca^2+^, and Na^+^ concentrations were assumed, in scenario “B,” K^+^ concentration was generated from the developed model, and constants Ca^2+^ and Na^+^ were assumed, in scenario “C,” Na^+^ concentration was generated from the developed model, and constants Ca^2+^ and K^+^ were assumed, in scenario “D,” Ca^2+^ concentration was generated from the developed model, and constant K^+^ and Na^+^ were assumed, and in scenario “E,” K^+^, Ca^2+^, and Na^+^ concentrations were generated from the developed models.

**Figure 2:**
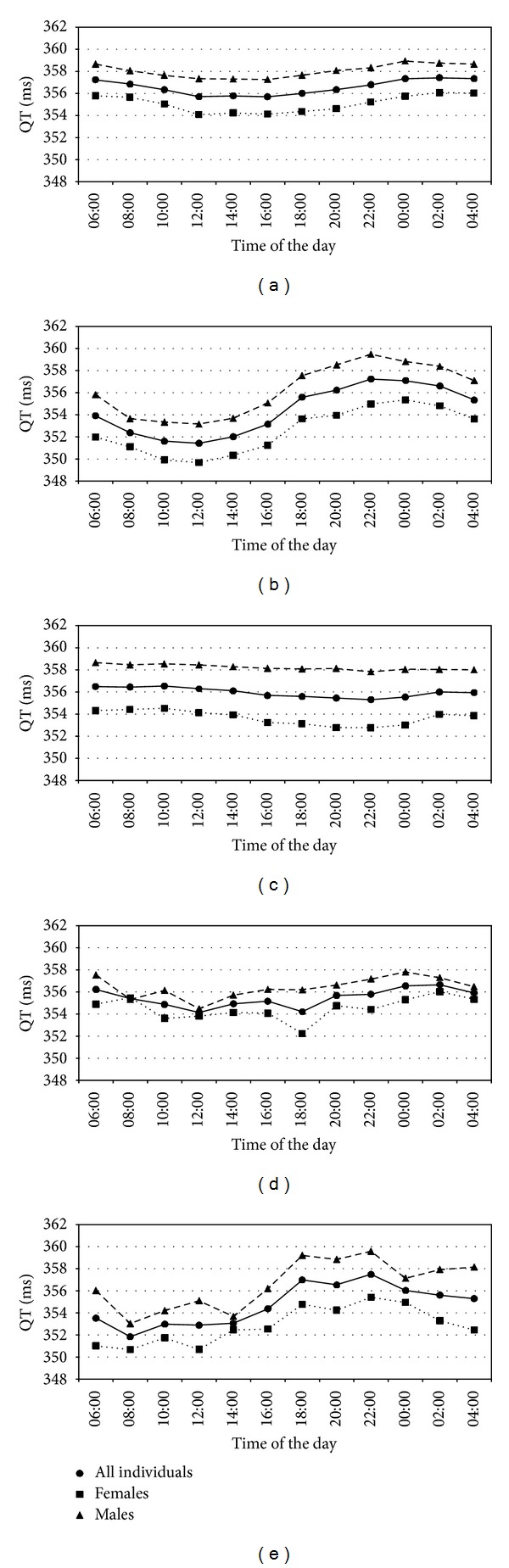
Mean QT values derived from the simulated scenarios.

**Figure 3:**
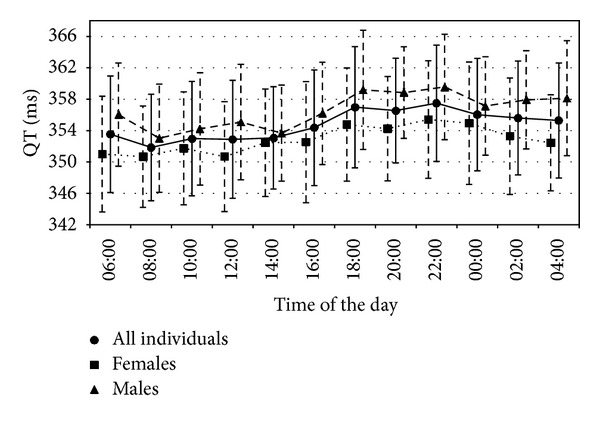
Mean and standard deviation of QT values derived only from the scenario “E.”

Assessment of the circadian variation of the pseudo-ECG derived QT length was the endpoint of the simulation study. Circadian changes, if present, could only result from the circadian ion concentration rhythmicity and circadian heart rate rhythmicity, with the latter enabled in all simulation scenarios, because the ToxComp platform in its current version does not include any other sources of circadian variability. Simulation results are presented in Figures 2 and 3. A near lack of the circadian variability was noted for the scenarios with sodium and calcium ions following proposed models, similar to the results for all ion concentrations set to constant values (scenarios A, C, and D, resp.), which was expected, except for sodium. Simulation results suggested a significant role of potassium in inducing circadian variability in QT (scenario B). What is important, the two scenarios where diurnal variation of the simulated QT was clearly present (scenarios B and E) also exhibited characteristics that were observed in the real (not simulated) clinical studies [47–49]; that is, QT value increases starting from the evening hours, reaching highest values very early in the morning (about 4 am). After that, QT value drops and remains relatively stable between 8 am and 4 pm when it again starts to rise. In all scenarios, a gender difference was observed in the simulated mean QT levels, which was consistent with finding that women have on average lower levels of electrolytes' concentration throughout the day.  Figure 3 presents more detailed information regarding the QT levels simulated in scenario E. Despite the fact that ionic models included sex-dependent dispersion, it may be seen that dispersion of QT values for both genders is very similar. It may be the case that the larger variability of potassium and sodium in females was compensated by the larger variability of calcium in males.

## 7. Discussion

In the first paragraph of this section, modelling limitations are described, and in the second paragraph, a brief indication of alternative uses of the developed models is given.

The stochastic structure of the developed models was based on small data sets. As a result, the dependence between consecutive concentrations was described by a simple autoregressive model. However, a real process may exhibit a far more sophisticated structure. Nevertheless, it should be noted that even this simple autoregressive process is believed to be a significant improvement upon a naive assumption of independence of consecutive concentrations. Also, due to the nature of the available data, the stochastic submodels were developed separately from the mean submodels (in principle, joint modelling should be preferred whenever possible, as it leads to a more efficient use of data). Also, the mix of the aggregated and individual data from different sources caused a major problem for the quantitative assessment of the model fit and model validation. Consequently, external validity of the simulated paths of ionic concentrations was graded by two domain experts who stated that the paths do not possess features that might disqualify them from the physiological point of view. The only raised concern regarded a high intrasubject variability of potassium and calcium, which may be a result of using AR process with short memory; however, as it was mentioned before, the data were not sufficient to support a more sophisticated autoregressive model. Further research is definitely needed to objectively resolve this issue. As it was justified in the main text, the modified Sennels' models were utilized for potassium and sodium. However, in Sennels, only male volunteers were included; therefore, the assumption of the gender independent structure of the circadian rhythm may potentially introduce a bias. Also, it should be reiterated that sodium and potassium models include a circadian rhythm but calcium does not, due to lack of contrary evidence. 

Current studies are aimed at establishing a connection between pathogenesis of cardiac diseases and circadian rhythms. Such a connection might be helpful in developing new chronotherapeutics and an early assessment of potential safety concerns. Circadian models of ions may be considered in simulations of drugs' activity, including their side effects, efficacy, and targeting ability. Also, there are suggestions that a synchronization of the cardiac clock with drug delivery strategies may bring benefits in terms of avoiding cardiotoxic events. Therefore, there is a need not only for studies presenting models useful for chronopharmaceutical research, but also concerning the potential use of chemical oscillators as biomarkers for new chronotherapeutics and chronopharmacological schedules [1, 2, 4, 50]. Additionally, it is possible that circadian models of ion levels may play a role in researching pharmacokinetics and pharmacodynamics of new chronotherapeutics and “old” drugs (e.g., *β*-blockers, calcium channel blockers), as it is the subject of numerous studies whether the time of the day when the medication is administered influences its effect or toxicity [2, 51].

## Supplementary Material

The electronic supplement contains a fully functional and ready to use implementation of models of serum potassium, sodium, and calcium concentrations described in the article along with a capability to simulate random paths of ionic concentrations.

## Figures and Tables

**Table 1:** Estimation results of mean concentration and standard deviation of concentration.

Ion	Sex	Number of subjects	Mean	Standard deviation (SD)
Potassium	Female	328	4.088	0.445
	Male	496	4.213	0.347
Sodium	Female	322	138.169	4.398
	Male	553	140.096	3.017
Calcium	Female	1783	2.313	0.136
	Male	475	2.418	0.199

**Table 2:** Estimation results for stochastic structure of ionic models.

Ion	Sex	SD of residuals	SD of residuals in AR(1) process	AR(1) coefficient
Potassium	Female	0.303	0.119	0.92
	Male	0.236	0.092	
Sodium	Female	1.866	0.371	0.98
	Male	1.280	0.255	
Calcium (logit scale)	Female	0.80	0.224	0.96
	Male	1.30	0.364	
