# Supplementary figures and images for: Circadian Models of Serum Potassium, Sodium, and Calcium Concentrations in Healthy Individuals and Their Application to Cardiac Electrophysiology Simulations at Individual Level
Source: Comput Math Methods Med. 2013 Sep 3;2013:429037. doi: 10.1155/2013/429037 (PMC3775438; doi:10.1155/2013/429037)

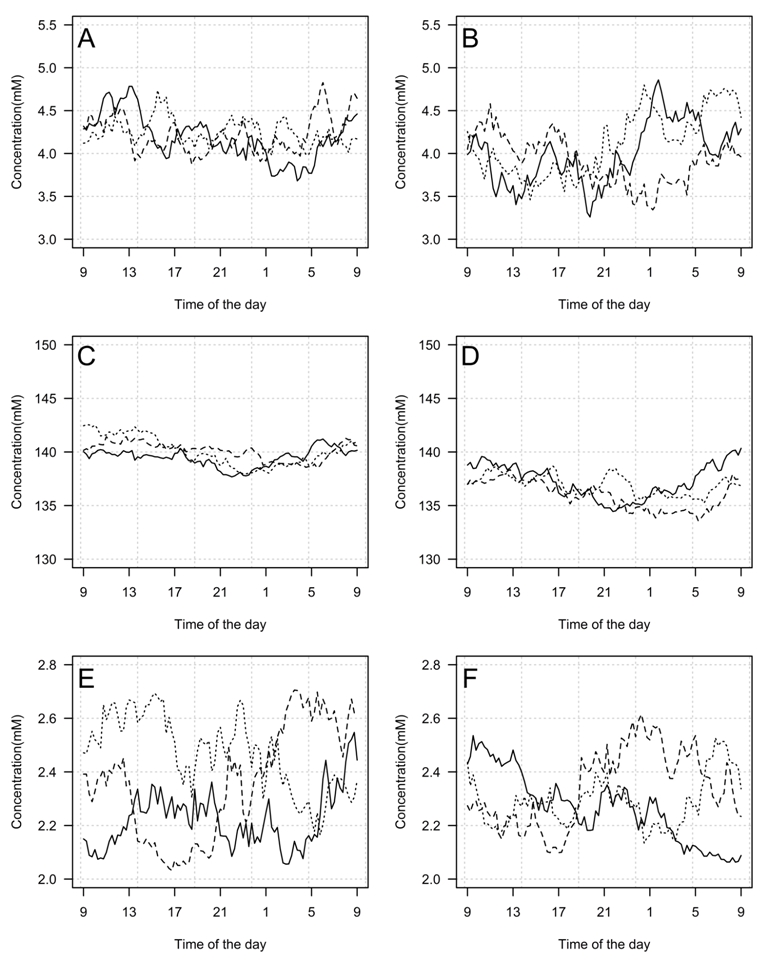

Supplement: Supplementary file 1 — The electronic supplement contains a fully functional and ready to use implementation of models of serum potassium, sodium, and calcium concentrations described in the article along with a capability to simulate random paths of ionic concentrations. [file 429037.f1.jpg]

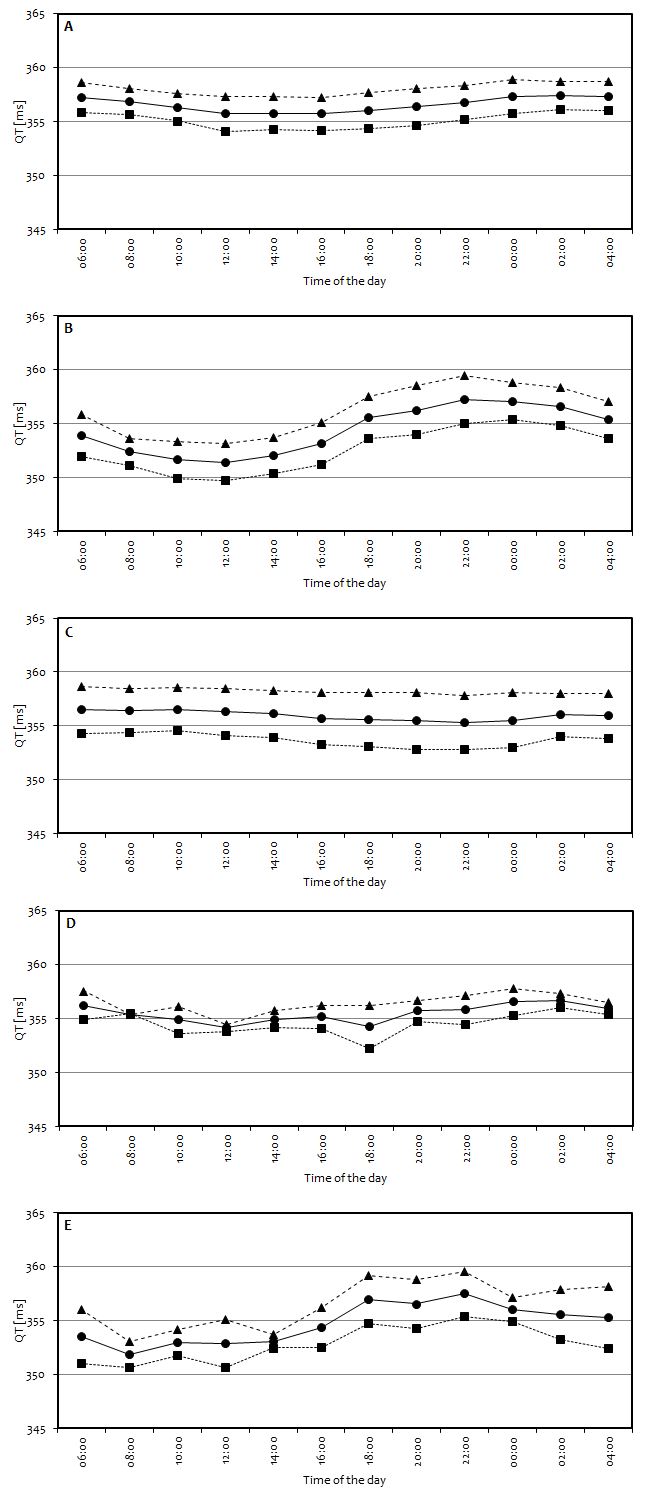

Supplement: Supplementary file 2 [file 429037.f2.jpg]

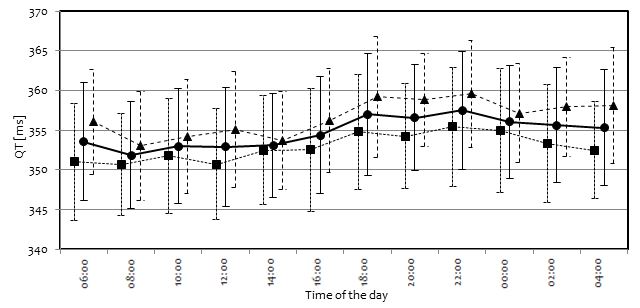

Supplement: Supplementary file 3 [file 429037.f3.jpg]
